# *ATP1A3* variants and slowly progressive cerebellar ataxia without paroxysmal or episodic symptoms in children

**DOI:** 10.1111/dmcn.14666

**Published:** 2020-09-07

**Authors:** Masayuki Sasaki, Noriko Sumitomo, Yuko Shimizu‐Motohashi, Eri Takeshita, Kenji Kurosawa, Kenjiro Kosaki, Kazuhiro Iwama, Takeshi Mizuguchi, Naomichi Matsumoto

**Affiliations:** ^1^ Department of Child Neurology National Center of Neurology and Psychiatry Tokyo Japan; ^2^ Division of Genetics Kanagawa Children’s Medical Center Yokohama Japan; ^3^ Center for Medical Genetics Keio University School of Medicine Tokyo Japan; ^4^ Department of Human Genetics Yokohama City University Graduate School of Medicine Yokohama Japan

## Abstract

A heterogeneous spectrum of clinical manifestations caused by mutations in *ATP1A3* have been previously described. Here we report two cases of infantile‐onset cerebellar ataxia, due to two different *ATP1A3* variants. Both patients showed slowly progressive cerebellar ataxia without paroxysmal or episodic symptoms. Brain magnetic resonance imaging revealed mild cerebellar cortical atrophy in both patients. Whole exome sequencing revealed a de novo heterozygous variant in *ATP1A3* in both patients. One patient had the c.460A>G (p.Met154Val) variant, while the other carried the c.1050C>A (p.Asp350Lys) variant. This phenotype was characterized by a slowly progressive cerebellar ataxia since the infantile period, which has not been previously described in association with *ATP1A3* variants or in *ATP1A3*‐related clinical conditions. Our report contributes to extend the phenotypic spectrum of *ATP1A3* mutations, showing paediatric slowly progressive cerebellar ataxia with mild cerebellar atrophy alone as an additional clinical presentation of *ATP1A3*‐related neurological disorders.

## Abstract 2

This article is commented on by Ng on pages 11‐12 of this issue.

**Abbreviations**
AHC: Alternating hemiplegia of childhood
CAPOS syndrome: Cerebellar ataxia, areflexia, pes cavus, optic atrophy, and sensory motor hearing loss

**What this paper adds**

- Novel *ATP1A3* variants are associated with slowly progressive cerebellar ataxia.
- No paroxysmal or episodic symptoms were found in our cases arising from *ATP1A3* variants.

The disease causing missense variants in *ATP1A3* was first identified in families with rapid‐onset dystonia parkinsonism in 2004, and since then several *ATP1A3*‐related disorders have been recognized.^1^, ^2^ Classic phenotypes include alternating hemiplegia of childhood (AHC)^3^ and rapid‐onset dystonia parkinsonism.^4^ More recently other phenotypes have been associated with variants in *ATP1A3*, such as cerebellar ataxia, areflexia, pes cavus, optic atrophy, and sensory motor hearing loss (CAPOS syndrome),^5^ relapsing encephalopathy with cerebellar ataxia^6^/fever‐induced paroxysmal weakness encephalopathy,^7^ early‐onset epileptic encephalopathy,^8^ rapid‐onset ataxia in childhood^9^ or adulthood,^10^ childhood‐onset schizophrenia,^11^ and autism spectrum disorders.^12^

Paroxysmal episodic symptoms such as transient tonic or flaccid hemiplegia, dystonia, tonic seizures, episodic cerebellar ataxia, and abnormal ocular movements are the most common symptoms in patients with *ATP1A3*‐related disorders^1^, ^2^ (Table 1), except for childhood‐onset schizophrenia or autism spectrum disorders. Thus, identifying these symptoms can be helpful in clinical practice to aid in the early diagnosis of *ATP1A3*‐related disorders. However, in intermittent periods between these paroxysmal symptoms, most patients with *ATP1A3*‐related disorders present with persistent neurological deficits such as hypotonia, motor delay, ataxia, nystagmus, cognitive and behavioural dysfunction, or involuntary movements such as dystonia or choreoathetosis.^1^ Interestingly, less severe *ATP1A3* phenotypes have not been reported to date. Here we report two cases of *ATP1A3*‐related neurological disorders with infantile‐onset slowly progressive cerebellar ataxia and mild or moderate intellectual disability, but without paroxysmal episodic signs or motor fluctuation.

**Table 1:** Main features reported in *ATP1A3*‐related disorders

	AHC^1^, ^2^, ^3^	RDP^1^, ^2^, ^4^	CAPOS^5^	RECA^6^/FIPWE^7^	ROA in childhood^9^ or adulthood^10^	Present cases
Main symptoms	Repeated attacks of hemiplegia that alternate in laterality Dystonic spells Seizure‐like episodes	Rapid‐onset of dystonia and parkinsonism Prominent bulbar findings	Cerebellar ataxia, areflexia, pes cavus, optic atrophy, and sensorineural hearing loss	Relapsing cerebellar ataxia and/or weakness	Rapid‐onset cerebellar ataxia	Slowly progressive cerebellar ataxia
Paroxysmal or episodic symptoms Frequency	Paroxysmal onset of hemiplegia Several times a month	Rapid‐onset dystonia Rarely repeated	Episodic cerebellar ataxia Less than once a year	Episodic onset ataxia/weakness Less than once a year	Rapid‐onset and stabilized ataxia Rarely repeated	No paroxysmal nor episodic symptoms
Cerebellar symptoms	Ataxia (slowly progressive in some cases)	Ataxia	Ataxia (recover or persistent)	Ataxia (stepwise progressive)	Ataxia (rapid‐onset, stabilized)	Ataxia (insidious onset)
Brain MRI findings	Cerebellar cortical atrophy (in some cases)	Normal	Normal	Normal	Cerebellar cortical atrophy	Cerebellar cortical atrophy

AHC, alternating hemiplegia of childhood; RDP, rapid‐onset dystonia parkinsonism; CAPOS, cerebellar ataxia, areflexia, pes cavus, optic atrophy, and sensorineural hearing loss; RECA, relapsing encephalopathy with cerebellar ataxia; FIPWE, fever‐induced paroxysmal weakness and encephalopathy; ROA, rapid‐onset ataxia; MRI, magnetic resonance imaging.

## CASE REPORT

Two adolescent (case 1: male, 15y; case 2: female, 12y) patients from the National Center of Neurology and Psychiatry in Tokyo were studied. Ataxia was assessed using the Scale for the Assessment and Rating of Ataxia (0–40). This study was approved by the ethical committee of the National Center of Neurology and Psychiatry. Written informed consent was obtained from the parents.

### Case 1

**Figure 1:**
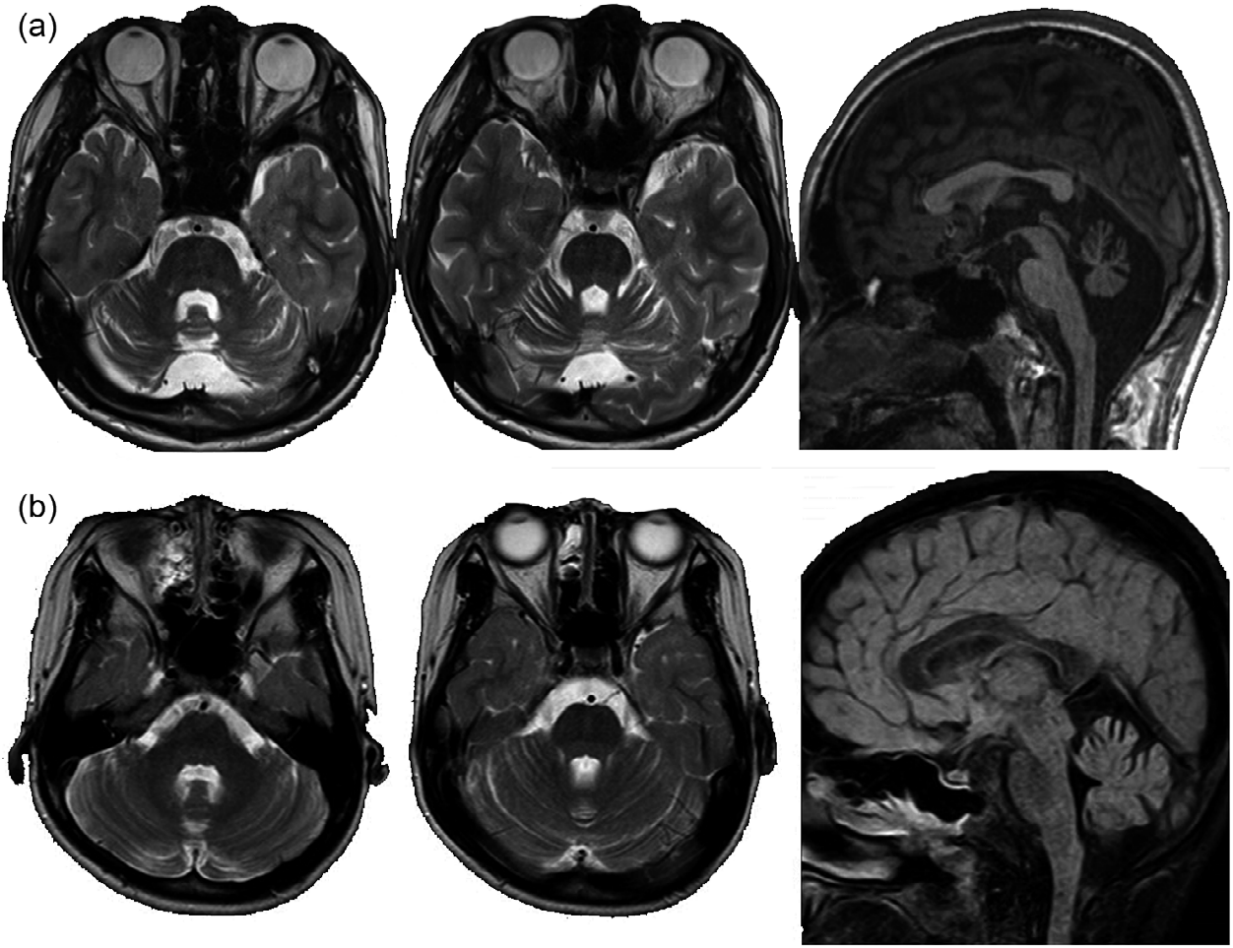
Brain magnetic resonance imaging (MRI) in case 1 (a) and Case 2 (b). (a) Brain MRI findings (axial; T2‐weighted images, coronal; short‐T1 inversion recovery, sagittal: T1) of case 1 (age 15y). Diffuse cerebellar cortical atrophy is seen predominantly in the vermis. No abnormal signal intensity is seen. (b) Brain MRI findings (axial; T2‐weighted images, sagittal; fluid‐attenuated inversion recovery) of case 2 (age 7y). Diffuse mild cerebellar cortical atrophy is seen. No abnormal signal intensity is seen.

Case 1 was born after an uneventful pregnancy from non‐consanguineous parents. He was able to smile at 2 months, hold his neck up at 3 months, sit without support at 8 months, walk unassisted at 24 months, and vocalize words at 22 months. He presented unstableness at 12 months in his sitting position. Walking was always unsteady with wide‐based gait. He communicated using several words slowly and unclearly. At 15 years, his ataxic symptoms gradually became worse and he often fell while walking; he was referred to our hospital at this stage. Based on the Wechsler Intelligence Scale for Children, his IQ was 40 and the neurological examination performed at 15 years indicated cerebellar ataxia including saccadic eye movement, ocular motor apraxia, dysarthria, dysdiadochokinesis, dysmetria, intention tremor, decomposition of movements, and wide‐based gait. Deep tendon reflexes were within the normal range. His Scale for the Assessment and Rating of Ataxia score was 20. He had no positive family history. He had no telangiectasia, pes cavus, or visual/hearing disabilities. There were no signs of episodic abnormal ocular movement, seizures, dystonic events, bulbar symptoms, autonomic dysfunction, or respiratory disturbances. Brain magnetic resonance imaging (MRI) revealed pure cerebellar cortical atrophy dominantly in the vermis (Fig. 1a).

### Case 2

Case 2 was born after an uneventful pregnancy from non‐consanguineous parents. She was able to hold her neck up at 5 months, sit without support at 15 months, walk unassisted at 2 years 11 months, and vocalize a word at 3 years. Her walking was always unsteady. At 7 years, she went to a hospital for unsteady walking, and brain MRI revealed a slight cerebellar cortical atrophy (Fig. 1b). Increased unsteadiness was reported when she had fever by viral infection. The unsteadiness gradually increased, and she was then referred to our hospital. Neurological examination revealed mild intellectual disability, saccadic eye movement and wide‐based gait, dysdiadochokinesis, dysmetria, and intention tremor. Normal deep tendon reflexes, visual acuity, and hearing ability were attested. Her Scale for the Assessment and Rating of Ataxia score was 12. She had no positive family history. She had no telangiectasia, pes cavus, or visual/hearing disabilities. There were no signs of episodic abnormal ocular movement, seizures, dystonic events, bulbar symptoms, autonomic dysfunction, or respiratory disturbances.

Both patients showed motor developmental delay, gradually progressive cerebellar ataxia, and mild intellectual disabilities.

## RESULTS

Blood tests including alpha‐fetoprotein, albumin, cholesterol, lactate/pyruvate, and cytosine‐thymine‐guanine repeats in spinocerebellar ataxia 1, 2, 3, and dentatorubral pallidoluysian atrophy disclosed no abnormalities in both patients. From the clinical and examination findings, ataxia telangiectasia, mitochondrial encephalopathies, neuronal ceroid lipofuscinosis, infantile neuroaxonal dystrophy, congenital disorders of glycosylation, spinocerebellar ataxia 1, 2, 3, and dentatorubral pallidoluysian atrophy were ruled out. Then, trio whole exome sequencing was carried out using previously described methodology.^13^

**Figure 2:**
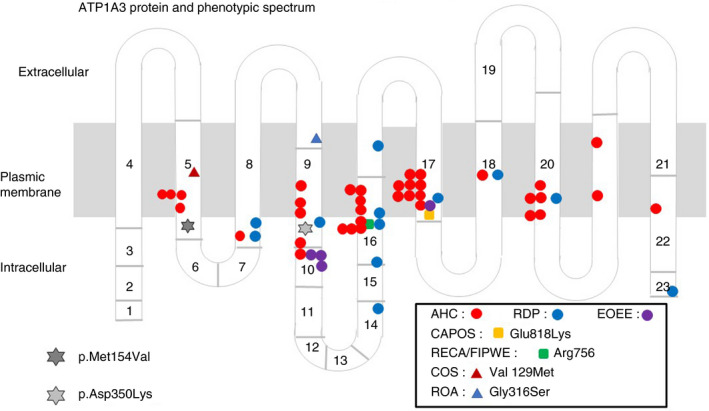
ATP1A3 protein and phenotypic spectrum. AHC, alternating hemiplegia of childhood; RDP, rapid‐onset dystonia‐parkinsonism; EOEE, early‐onset epileptic encephalopathy; CAPOS, cerebellar ataxia, areflexia, pes cavus, optic atrophy, and **s**ensorineural hearing loss; RECA/FIPWE, relapsing encephalopathy with cerebellar ataxia/fever‐induced paroxysmal weakness and encephalopathy; COS, childhood‐onset schizophrenia; ROA, rapid‐onset ataxia.

Variants in *ATP1A3* (NM. 152296.5) were identified by whole exome sequencing: c.460A>G: p.(Met154Val) in case 1 and c.1050C>A: p.(Asp350Lys) in case 2. These variants were confirmed by Sanger sequencing in the patients and their parents, and were found to be heterozygous de novo variants. These variants have not been reported previously, and computed analysis tools (SIFT, Polyphen2) predicted the likely pathogenic in each. Both variants are located on the intracellular region of Na^+^/K^+^‐ATPase α3 subunit (Fig. 2).

Whole exome sequencing excluded ataxia telangiectasia, ataxia‐oculomotor apraxia 1/2, spinocerebellar ataxia 5/13/29, and many other autosomal dominant or recessive cerebellar ataxia disorders in childhood.

## DISCUSSION

The purpose of this study was to advance the understanding of phenotypes associated with variants in *ATP1A3*. Specifically, we reported two cases with symptoms that have not been previously associated with variants in *ATP1A3*. These symptoms include infantile‐onset of slowly progressive pure cerebellar ataxia and mild to moderate intellectual disability, which have not been previously reported in patients with *ATP1A3*‐related disorders (Table 1). Thus, these two cases did not show any characteristic symptoms of CAPOS^5^ syndrome, relapsing encephalopathy with cerebellar ataxia^6^/fever‐induced paroxysmal weakness encephalopathy,^7^ nor any paroxysmal symptoms usually seen in AHC^3^ and rapid‐onset dystonia parkinsonism.^4^

Brain MRI in both patients revealed pure mild cerebellar cortical atrophy. In *ATP1A3*‐related disorders, brain MRI abnormalities are not generally detected. However, as we previously reported,^14^ some patients with typical AHC show mild cerebellar cortical atrophy in adulthood. The cerebellar atrophy detected by brain MRI in both cases of the current study is similar to the atrophy previously reported in typical patients with AHC and the c.2401G>A (p.Asp801Asn) variant or c.2423C>T (p.Pro808Leu) variant in *ATP1A3*.^14^ Therefore, pure mild cerebellar cortical atrophy may be a trait that can help to identify progressive cerebellar ataxia cases of *ATP1A3*‐related neurological disorders. The mechanisms of underlaying this relation are unknown, as the Na^+^/K^+^‐ATPase α3 subunit is ubiquitously expressed in the brain, including the cerebellar cortex,^15^ which does not explain our current finding of pure cerebellar atrophy.

Recently, some adult patients with rapid‐onset ataxia were reported,^10^ and patients with childhood‐onset ataxia‐dominant with *ATP1A3* variants were also reported.^9^ However, patients with infantile‐onset and pure slowly progressive cerebellar ataxia caused by *ATP1A3* variants without any paroxysmal symptoms have not been reported. Currently, some phenotype/genotype correlations have been described. For instance, CAPOS syndrome is caused by p.Glu818Lys mutation^5^ and relapsing encephalopathy with cerebellar ataxia^6^/fever‐induced paroxysmal weakness encephalopathy^7^ is caused by p.Arg756 Ser/His/Cys mutation. The mutations which cause AHC are mostly different from those causing rapid‐onset dystonia parkinsonism. In AHC, a correlation between a relatively severe phenotype with the genotype of p.Glu815Lys, and a moderate phenotype with the genotype of p.Asp801Asn and p.Gln947Arg has been reported.^3^ In the present report, we describe for the first time two *ATP1A3* variants, namely p.Met154Val (case 1) and p.Asp350Lys (case 2). These are both located in the intracellular cytoplasmic region of the Na^+^/K^+^‐ATPase, but further research is needed to clarify the potential modifications affecting the regulation of Na^+^/K^+^‐ATPase.

Although based on only two patients, we believe that the symptoms reported, namely infantile‐onset and slowly progressive cerebellar ataxia, could be a new subtype of *ATP1A3*‐related neurological disorders. Overall, the findings have important implications for the diagnosis of spinocerebellar ataxia.
